# A genome assembly of the American black bear, *Ursus americanus*, from California

**DOI:** 10.1093/jhered/esae037

**Published:** 2024-07-15

**Authors:** Megan A Supple, Merly Escalona, Jillian Adkins, Michael R Buchalski, Nicolas Alexandre, Ruta M Sahasrabudhe, Oanh Nguyen, Samuel Sacco, Colin Fairbairn, Eric Beraut, William Seligmann, Richard E Green, Erin Meredith, Beth Shapiro

**Affiliations:** Department of Ecology and Evolutionary Biology, University of California, Santa Cruz, CA, United States; Howard Hughes Medical Institute, University of California, Santa Cruz, CA, United States; Department of Biomolecular Engineering, University of California, Santa Cruz, CA, United States; Wildlife Forensic Lab, Law Enforcement Division, California Department of Fish and Wildlife, Sacramento, CA, United States; Wildlife Genetics Research Unit, Wildlife Health Laboratory, California Department of Fish and Wildlife, Sacramento, CA, United States; Department of Ecology and Evolutionary Biology, University of California, Santa Cruz, CA, United States; Howard Hughes Medical Institute, University of California, Santa Cruz, CA, United States; DNA Technologies and Expression Analysis Core Laboratory, Genome Center, University of California, Davis, CA, United States; DNA Technologies and Expression Analysis Core Laboratory, Genome Center, University of California, Davis, CA, United States; Department of Ecology and Evolutionary Biology, University of California, Santa Cruz, CA, United States; Department of Ecology and Evolutionary Biology, University of California, Santa Cruz, CA, United States; Department of Ecology and Evolutionary Biology, University of California, Santa Cruz, CA, United States; Department of Ecology and Evolutionary Biology, University of California, Santa Cruz, CA, United States; Department of Biomolecular Engineering, University of California, Santa Cruz, CA, United States; Wildlife Forensic Lab, Law Enforcement Division, California Department of Fish and Wildlife, Sacramento, CA, United States; Department of Ecology and Evolutionary Biology, University of California, Santa Cruz, CA, United States; Howard Hughes Medical Institute, University of California, Santa Cruz, CA, United States

**Keywords:** California Conservation Genomics Project, CCGP, Conservation Genomics, wildlife management

## Abstract

The American black bear, *Ursus americanus*, is a widespread and ecologically important species in North America. In California, the black bear plays an important role in a variety of ecosystems and serves as an important species for recreational hunting. While research suggests that the populations in California are currently healthy, continued monitoring is critical, with genomic analyses providing an important surveillance tool. Here we report a high-quality, near chromosome-level genome assembly from a *U. americanus* sample from California. The primary assembly has a total length of 2.5 Gb contained in 316 scaffolds, a contig N50 of 58.9 Mb, a scaffold N50 of 67.6 Mb, and a BUSCO completeness score of 96%. This *U. americanus* genome assembly will provide an important resource for the targeted management of black bear populations in California, with the goal of achieving an appropriate balance between the recreational value of black bears and the maintenance of viable populations. The high quality of this genome assembly will also make it a valuable resource for comparative genomic analyses among black bear populations and among bear species.

## Introduction

**Fig. 1.**
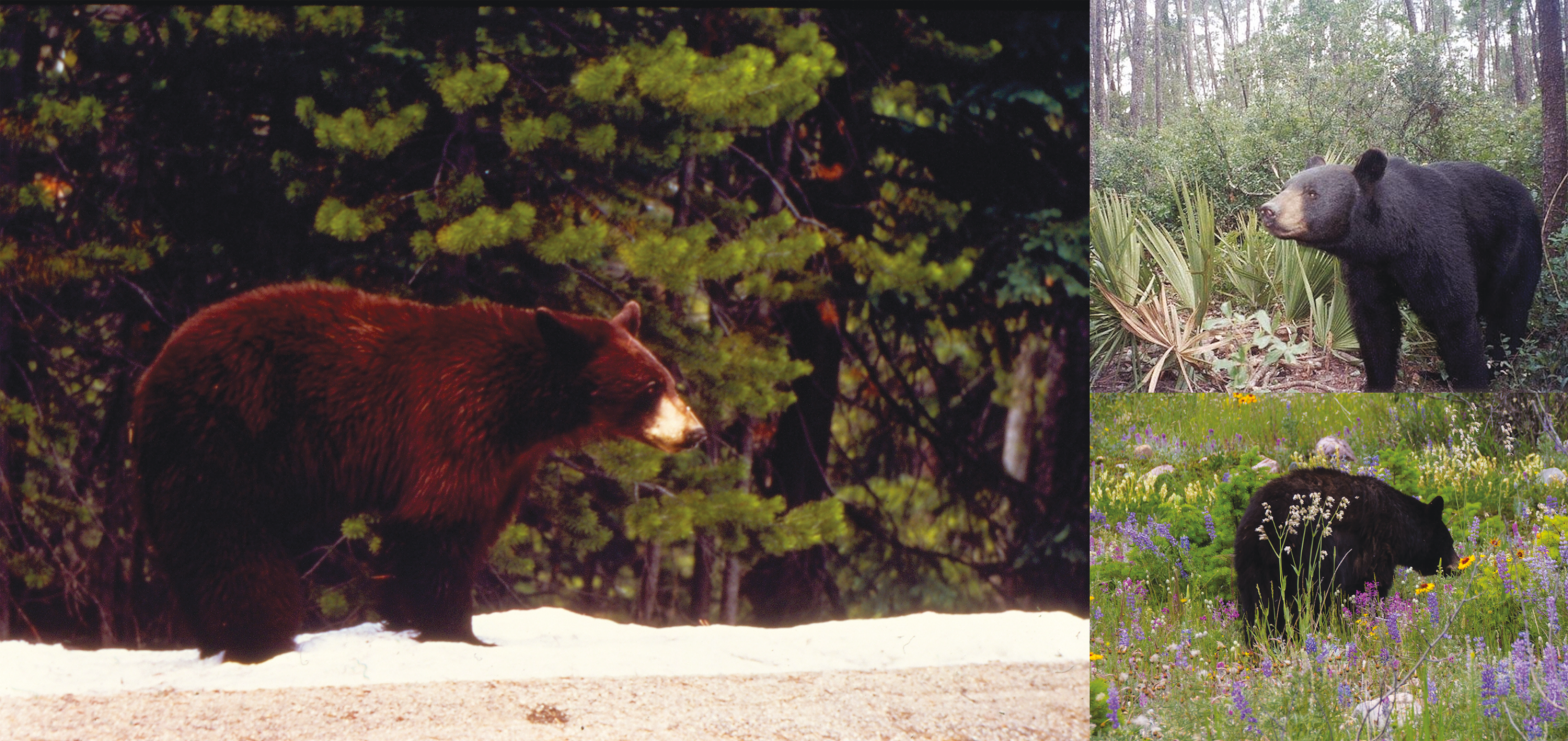
The American black bear, *Ursus americanus*, is a widespread species that can be found in a variety of habitats, from dense forests to open grasslands. Photos from California Department of Fish and Wildlife (left, CC BY 2.0), Florida Fish and Wildlife Conservation Commission (top right, public domain), and David Wasserman (bottom right, CC BY-SA 4.0).

The American black bear, *Ursus americanus*, is an ecologically and economically important species in North America (Fig. 1). Historically, black bears were widely distributed, but loss of habitat has restricted that range, particularly in the United States (Pelton et al. 1999). In California, the species plays a critical role in many ecosystems, while also serving as an important species for recreational hunting (California Department of Fish and Game 1998). While research suggests that the California populations are currently healthy, continued monitoring is critical to developing targeted management plans in order to achieve an appropriate balance between the recreational value of black bears and the maintenance of viable populations across the state (California Department of Fish and Game 1998).

Genomic resources, including high-quality genome assemblies, will provide valuable tools for the assessment of black bear populations. Genomic analyses will enable the development of population-specific management strategies by assessing population connectivity, inbreeding depression, and local adaptation. The results of these analyses will aid managers in maintaining healthy black bear populations across their range. This genome, generated from a sample from California, will be instrumental in understanding genetic variation unique to populations in the western United States and can also be used in pangenomic analyses with existing assemblies to better represent the diversity of black bears throughout their native range. There are publicly available genome assemblies from two samples, both from the eastern United States. One is contig-level (NCBI accession GCF_020975775.1); the other is scaffold-level (GCA_003344425.1, Srivastava et al. 2019). Multiple reference genomes from divergent lineages enable the identification of structural variants, which may play a critical role in local adaptation and population health.

High-quality genome assemblies will also enable comparative genomics analyses across bear species. Recent advances in multiple reference genome alignment have enabled the discovery of genetic characteristics important to species conservation (Wilder et al. 2023), as well as the evolutionary innovations unique to various lineages (Christmas et al. 2023). Ongoing efforts to generate high-quality genome assemblies for all extant bear lineages will enable the identification of deleterious and adaptive genetic variation, both within the lineage and at broader taxonomic levels.

Here we report a high-quality, near chromosome-level genome assembly generated from a California black bear as part of the California Conservation Genomics Project (CCGP; Shaffer et al. 2022). This genome assembly will be a valuable resource for management of the black bears across California and the rest of North America.

## Methods

### Biological materials

We captured and sedated an adult male black bear (L20-20) for relocation in September 2020 at Kings Beach, Placer County, California (39.2377°N, 120.0266°W). California Department of Fish and Wildlife (CDFW) staff captured the bear under the department’s jurisdiction as the trustee for wildlife management in the state of California, CA Fish & Game Code § 1802 (2015). While the bear was sedated, CDFW staff collected a whole-blood sample into a tube containing EDTA.

### DNA extraction

We isolated high molecular weight (HMW) genomic DNA (gDNA) from the whole blood sample. We added 3 ml of RBC lysis solution (Qiagen, Germany; Cat # 158445) to 1 ml of whole blood and incubated the reaction at room temperature for 5 min. We centrifuged the sample at 2,000 x *g* for 5 min to pellet white blood cells. We discarded the supernatant and added 2 ml of lysis buffer containing 100 mM NaCl, 10 mM Tris-HCl pH 8.0, 25 mM EDTA, 0.5% (w/v) SDS, and 100 µg/ml Proteinase K to the cell pellet. We incubated the reaction at room temperature for a few hours until the solution was homogenous. We then treated the lysate with 20 µg/ml RNase A at 37 °C for 30 min and cleaned with equal volumes of phenol/chloroform using phase lock gels (Quantabio, Beverly, MA; Cat # 2302830). We precipitated the DNA by adding 0.4× volume of 5M ammonium acetate and 3× volume of ice-cold ethanol. We washed the DNA pellet twice with 70% ethanol and resuspended it in an elution buffer (10 mM Tris, pH 8.0). We assessed the purity of gDNA using a NanoDrop ND-1000 spectrophotometer and observed a 260/280 ratio of 1.85 and a 260/230 ratio of 2.13. We quantified the DNA yield at 15 µg with a Qbit 2.0 Fluorometer (Thermo Fisher Scientific, Waltham, MA). We verified the integrity of the HMW gDNA on a Femto pulse system (Agilent Technologies, Santa Clara, CA), confirming that 70% of the DNA was in fragments over 100 kb.

### PacBio HiFi library preparation and sequencing

We constructed a HiFi SMRTbell library using the SMRTbell Express Template Prep Kit v2.0 (Pacific Biosciences [PacBio], Menlo Park, CA; Cat. #100-938-900) according to the manufacturer’s instructions. We sheared HMW gDNA to a target DNA size distribution of 15 to 20 kb using Diagenode’s Megaruptor 3 system (Diagenode, Belgium; Cat. B06010003) and then concentrated the sheared DNA using 0.45× of AMPure PB beads (PacBio; Cat. #100-265-900). We then processed the DNA through a series of enzymatic reactions: removal of single-strand overhangs at 37 °C for 15 min, DNA damage repair at 37 °C for 30 min, end repair and A-tailing at 20 °C for 10 min and 65 °C for 30 min, ligation of overhang adapters v3 at 20 °C for 60 min followed by 65 °C for 10 min to inactivate the ligase, and nuclease treatment at 37 °C for 1 h. We then purified and concentrated the SMRTbell library with 0.45× Ampure PB beads for size selection using the BluePippin/PippinHT system (Sage Science, Beverly, MA; Cat #BLF7510/HPE7510) to collect fragments greater than 9 kb, with a resulting average fragment size of 16 kb. We sequenced the HiFi SMRTbell library at UC Davis DNA Technologies Core (Davis, CA) using three SMRT Cell 8M trays (PacBio, Cat #101-389-001), Sequel II sequencing chemistry 2.0, and 30-h movies for each cell on a PacBio Sequel II sequencer.

### Omni-C library preparation and sequencing

We prepared an Omni-C library from whole blood (ID:AMBB2020-038-001) using a Dovetail Omni-C Kit (Dovetail Genomics, Scotts Valley, CA) according to the manufacturer’s protocol with slight modifications. We crosslinked the chromatin in the nucleus, digested the chromatin with DNase I, repaired chromatin ends and ligated a biotinylated bridge adapter to the repaired ends, reversed the crosslinks, and purified the DNA. We treated purified DNA to remove biotin that was not internal to ligated fragments. We generated a short-read sequencing library using an NEB Ultra II DNA Library Prep kit (New England Biolabs, Ipswich, MA) with an Illumina-compatible y-adaptor. We captured biotin-containing fragments using streptavidin beads. We split the post-capture product into two replicates prior to polymerase chain reaction (PCR) enrichment to preserve library complexity, with each replicate receiving a unique dual index. We sequenced the library at the Vincent J. Coates Genomics Sequencing Laboratory (Berkeley, CA) on the Illumina NovaSeq 6000 platform with an S4 flow cell (Illumina, San Diego, CA).

### Nuclear genome assembly

We assembled the genome of *U. americanus* following the CCGP assembly pipeline version 4.0 (https://github.com/ccgproject/ccgp_assembly). Table 1 lists the software and non-default parameters used in the assembly. First, we removed the remnant adapter sequences from the PacBio HiFi dataset using HiFiAdapterFilt (Sim et al. 2022). We then generated the initial, partially phased, diploid assembly using HiFiasm (Cheng et al. 2022) in Hi-C mode using the adapter-trimmed PacBio HiFi reads and the Omni-C data. Next, we aligned the Omni-C data to the primary assembly following the Arima Genomics Mapping Pipeline (https://github.com/ArimaGenomics/mapping_pipeline) and then scaffolded it with SALSA (Ghurye et al. 2017, 2019).

**Table 1.** Assembly pipeline and software used.

Assembly step	Software and non-default options	Version	References
Filtering PacBio HiFi adapters	HiFiAdapterFilt	Commit 64d1c7b	Sim et al. (2022)
K-mer counting	Meryl (*k* = 21)	1	https://github.com/marbl/meryl
Estimation of genome size and heterozygosity	GenomeScope	2	Ranallo-Benavidez et al. (2020)
De novo assembly (contiging)	HiFiasm (HiC Mode, –primary, output hic.p_ctg, hic.a_ctg)	0.16.1-r375	Cheng et al. (2022)
Scaffolding			
Omni-C data alignment	Arima Genomics Mapping Pipeline	Commit 2e74ea4	https://github.com/ArimaGenomics/mapping_pipeline
Arima Genomics Mapping Pipeline (AGMP)	BWA-MEM	0.7.17-r1188	Li (2013)
	samtools	1.11	Danecek et al. (2021)
	filter_five_end.pl (AGMP)	Commit 2e74ea4	https://github.com/ArimaGenomics/mapping_pipeline
	two_read_bam_combiner.pl ((AGMP))	Commit 2e74ea4	https://github.com/ArimaGenomics/mapping_pipeline
	picard	2.27.5	https://broadinstitute.github.io/picard/
Omni-C Scaffolding	SALSA (-DNASE, -i 20, -p yes)	2	Ghurye et al. (2017, 2019)
Omni-C Contact map generation			
Short-read alignment	BWA-MEM (-5SP)	0.7.17-r1188	Li (2013)
SAM/BAM processing	samtools	1.11	Danecek et al. (2021)
SAM/BAM filtering	pairtools	0.3.0	Open2C et al. (2023)
Pairs indexing	pairix	0.3.7	Lee at al. (2022)
Matrix generation	cooler	0.8.10	Abdennur and Mirny (2020)
Matrix balancing	hicExplorer (hicCorrectmatrix correct --filterThreshold -2 4)	3.6	Ramírez et al. (2018)
Contact map visualization	HiGlass	2.1.11	Kerpedjiev et al. (2018)
	PretextMap	0.1.4	https://github.com/wtsi-hpag/PretextView
	PretextView	0.1.5	https://github.com/wtsi-hpag/PretextMap
	PretextSnapshot	0.0.3	https://github.com/wtsi-hpag/PretextSnapshot
Manual curation tools	Rapid curation pipeline (Wellcome Trust Sanger Institute, Genome Reference Informatics Team)	Commit 7acf220c	https://gitlab.com/wtsi-grit/rapid-curation
Genome quality assessment			
Basic assembly metrics	QUAST (--est-ref-size)	5.0.2	Gurevich et al. (2013)
Assembly completeness	BUSCO (-m geno, -l mammalia)	5.0.0	Manni et al. (2021)
	Merqury	2020-01-29	Rhie et al. (2020)
Contamination screening			
Local alignment tool	BLAST + (-db nt, -outfmt “6 qseqid staxids bitscore std,” -max_target_seqs 1, -max_hsps 1, -evalue 1e-25)	2.10	Camacho et al. (2009)
General contamination screening	BlobToolKit (PacBIo HiFi Coverage, NCBI Taxa ID = 9643, BUSCODB = mammalia)	2.3.3	Challis et al. (2020)
Mitochondrial assembly			
Mitochondrial genome assembly	MitoHiFi (-r, -p 90, -o 1)	2.2	Uliano-Silva et al. (2023)
Synteny analysis			
Sequence alignment tool	mummer (nucmer)	3.1	Kurtz et al. (2004)

We manually curated the primary haplotype by analyzing its Omni-C contact map. To generate the contact map, we aligned the Omni-C data with BWA-MEM (Li 2013), identified ligation junctions, and generated Omni-C pairs (Lee et al. 2022) using pairtools (Open2C et al. 2023). We generated a multi-resolution Omni-C matrix with cooler (Abdennur and Mirny 2020) and balanced it with hicExplorer (Ramírez et al. 2018). We used HiGlass (Kerpedjiev et al. 2018) and the PretextSuite (https://github.com/wtsi-hpag/PretextView; https://github.com/wtsi-hpag/PretextMap; https://github.com/wtsi-hpag/PretextSnapshot) to visualize the contact map in order to identify and break contigs at major misassemblies and misjoin locations. We modified the assembly using the Rapid Curation pipeline (https://gitlab.com/wtsi-grit/rapid-curation). Lastly, we checked for contamination using the BlobToolKit Framework (Challis et al. 2020).

We identified the X chromosome in our assembly using synteny with the domestic dog genome. We used Nucmer (Kurtz et al. 2004) to align a domestic dog X chromosome (NCBI RefSeq GCF_011100685.1, scaffold NC_049260.1) to our assembly and examined hits longer than 10 kb with greater than 80% identity. We also attempted to identify the Y chromosome in our assembly using the same process with a domestic dog Y chromosome (NCBI GenBank KP081776.1).

### Genome assembly assessment

We generated k-mer counts from the adapter-trimmed PacBio HiFi reads using Meryl (https://github.com/marbl/meryl). We used these k-mer counts in GenomeScope2.0 (Ranallo-Benavidez et al. 2020) to estimate genome features, including genome size, heterozygosity, and repeat content. To obtain general contiguity metrics, we ran QUAST (Gurevich et al. 2013). To evaluate genome quality and functional completeness we used BUSCO (Manni et al. 2021) with the Mammalia ortholog database (mammalia_odb10), which contains 9,226 genes. We assessed base-level accuracy and k-mer completeness using Merqury (Rhie et al. 2020) with the previously generated Meryl database. We further estimated genome assembly accuracy using a frameshift analysis of the BUSCO gene set, as described in Korlach et al. (2017). We determined the size of the phased blocks based on the size of the contigs generated by HiFiasm in HiC mode. Following the quality metric nomenclature established by Rhie et al. (2021), we calculated the genome quality code *x*.*y*.*P*.*Q*.*C*, where, *x* = log10[contig NG50]; *y* = log10[scaffold NG50]; *P* = log10 [phased block NG50]; *Q* = Phred base accuracy QV (quality value); *C* = % genome represented by the first “*n*” scaffolds, following a karyotype of 2*n* = 74 (Nash and O’Brien 1987). We calculated these quality code metrics for the primary assembly.

We compared genome statistics for our assembly (mUrsAme1) to two other black bear genome assemblies available: ASM334442v1 (NCBI Genome: GCA_003344425.1) and gsx_jax_bbear_1 (NCBI RefSeq GCF_020975775.1). We generated the contiguity metrics using QUAST and the functional completeness metrics using BUSCO with the Mammalian ortholog database.

### Mitochondrial genome assembly

We assembled the mitochondrial genome of *U. americanus* from the PacBio HiFi reads using the reference-guided pipeline MitoHiFi (Uliano-Silva et al. 2023). We used an existing mitochondrial sequence of *U. americanus* (NCBI:AF303109.1; Delisle and Strobeck 2002) as the starting reference sequence. We searched for matches of the resulting mitochondrial assembly sequence in the nuclear genome assembly using BLAST+ (Camacho et al. 2009) and filtered out contigs and scaffolds from the nuclear genome assembly with a sequence identity >99% and size smaller than the mitochondrial assembly sequence. We annotated the mitochondrial genome using MitoFinder (Allio et al. 2020).

## Results

**Fig. 2.**
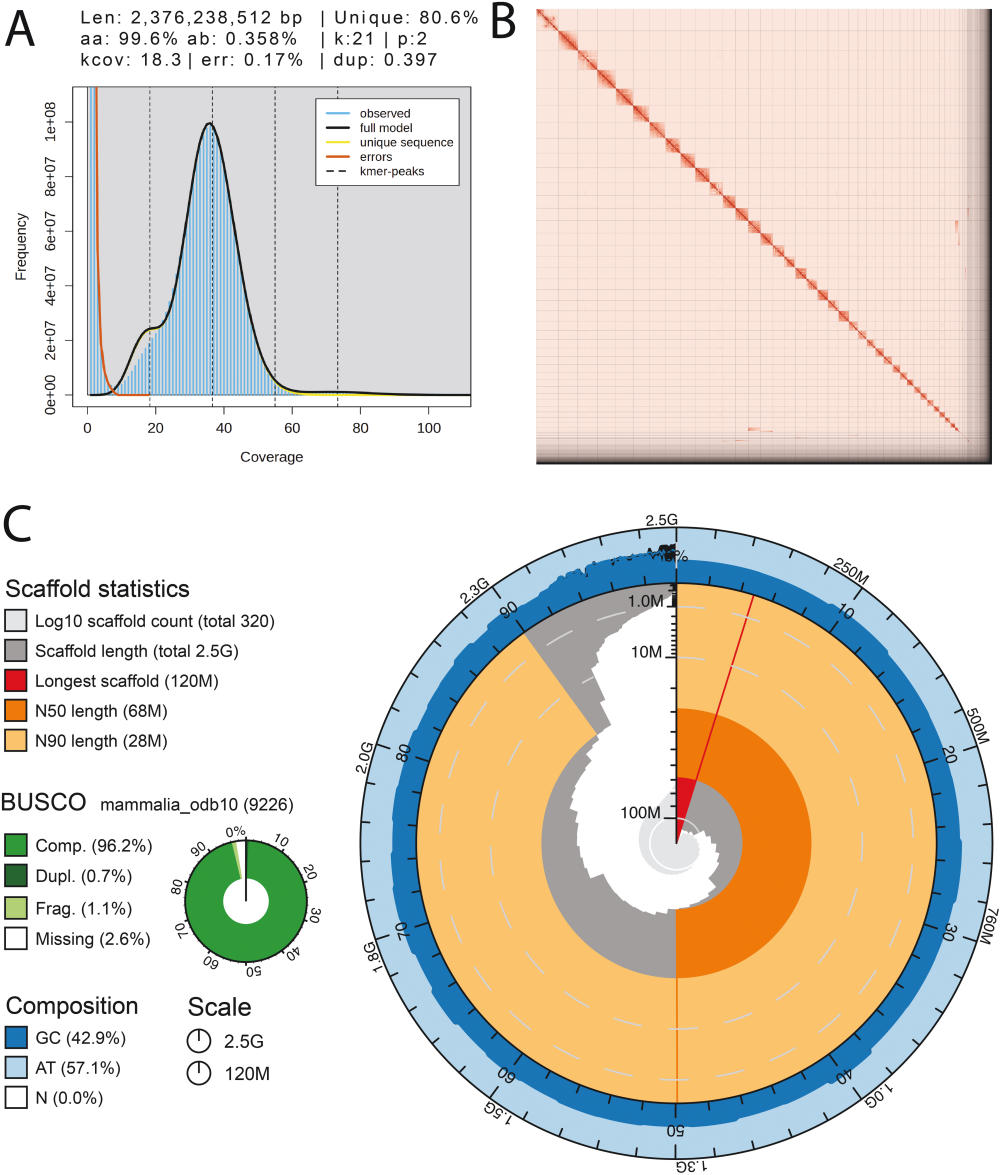
Visualization of assembly metrics. (A) K-mer frequencies from the adapter-trimmed PacBio HiFi data used to estimate genome size, sequencing error rate, and heterozygosity. The main peak at ~37× coverage corresponds to homozygous regions of the genome, while the slight peak at ~18× corresponds to heterozygous regions of the genome. The peak around zero corresponds to probable sequencing errors. (B) The omni-C contact map for the primary assembly after manual curation shows the 3D organization of the genome, with darker areas indicating closer proximity. (C) Snail plot displaying assembly metrics for the primary assembly.

The Omni-C library generated 130.9 million read pairs and the PacBio HiFi library generated 6.1 million reads. The PacBio HiFi sequences yielded ~38× genome coverage and had an N50 read length of 15,293 bp; a minimum read length of 43 bp; a mean read length of 14,915 bp; and a maximum read length of 52,231 bp (see Supplementary Fig. S1 for read length distribution). Based on the PacBio HiFi data, Genomescope 2.0 estimated a genome size of 2.37 Gb, a 0.17% sequencing error rate, and 0.358% heterozygosity. The k-mer spectrum shows a bimodal distribution with a major coverage peak at ~37× coverage and a minor coverage peak at ~18× coverage (Fig. 2A).

The final genome assembly (mUrsAme1) consists of two partially phased haplotypes. Both assemblies are similar in size, but not equal to the estimated genome size from GenomeScope2.0, as has been observed in other taxa (see Pflug et al. 2020, for example). The primary assembly (mUrsAme1.0.p) consists of 316 scaffolds spanning 2.52 Gb with a contig N50 of 58.85 Mb, a scaffold N50 of 67.55 Mb, the largest contig size of 107.13 Mb, and the largest scaffold size of 122.37 Mb. Given the level of fragmentation of the alternate assembly, we kept it as a contig-level assembly. The alternate assembly (mUrsAme1.0.a) consists of 77,310 contigs spanning 2.88 Gb with a contig N50 of 60.74 kb and the largest contig size of 831.37 kb. The fragmentation of the alternate assembly is likely due to the low heterozygosity of the sampled individual because the alternate assembly represents heterozygous regions of the genome.

The primary assembly has a BUSCO completeness score for the Mammalia gene set of 96.30%, a base pair QV of 63.01, k-mer completeness of 98.18%, and a frameshift indel QV of 43.13. The alternate assembly has a BUSCO completeness score for the Mammalia gene set of 62.6%, a base pair QV of 56.97, a k-mer completeness of 75.54%, and a frameshift indel QV of 42.81.

During manual curation, we made 11 joins and 1 break on the primary assembly based on the initial Omni-C contact map. We filtered out 4 contigs corresponding to mitochondrial contamination, one from the primary assembly and 3 from the alternate assembly. No further contigs were removed. The Omni-C contact map for the final primary assembly shows a highly contiguous assembly (Fig. 2B). Assembly statistics are reported in Table 2 and represented graphically in Fig. 2C. We have deposited the genome assembly on NCBI GenBank (see Table 2 and Data Availability for details).

**Table 2.** Assembly statistics and data availability.

Bio Projects and Vouchers	CCGP NCBI BioProject			PRJNA720569			
	Genera NCBI BioProject			PRJNA765883			
	Species NCBI BioProject			PRJNA777227			
	NCBI BioSample			SAMN29046565			
	Specimen identification			L20-20			
	NCBI Genome accessions			**Primary**		**Alternate**	
	Assembly accession			JANIGQ000000000		JANIGR000000000	
	Genome sequences			GCA_024610735.1		GCA_024610745.1	
Genome Sequence	PacBio HiFi reads		Run	1 PACBIO_SMRT (Sequel II) run:			
				6.1M spots, 90.4G bases, 48.9G bytes			
			Accession	SRX17388741			
	Omni-C Illumina reads		Runs	2 ILLUMINA (Illumina NovaSeq 6000) runs:			
				130.9M spots, 39.5G bases, 13.9G bytes			
			Accessions	SRX23638327, SRX23638328			
Genome Assembly Quality Metrics	Assembly identifier (Quality code^a^)				mUrsAme1(7.7.P7.Q58.C91)		
	HiFi Read coverage^b^			38.02×			
				**Primary**		**Alternate**	
	Number of contigs			339		77,310	
	Contig N50 (bp)			58,859,121		43,280	
	Contig NG50^b^				59,189,856	60,742	
	Longest Contigs			107,133,695		831,372	
	Number of scaffolds			316		77,310	
	Scaffold N50			67,550,933		43,280	
	Scaffold NG50^b^				68,367,985	60,742	
	Largest scaffold			122,379,270		831,372	
	Size of final assembly			2,524,264,886		2,885,111,500	
	Phased block NG50^b^				59,189,856		60,747
	Gaps per Gbp (# Gaps)			9 (22)		0 (0)	
	Indel QV (Frame shift)			43.1369853		42.81941933	
	Base pair QV			63.0115		56.9775	
				Full assembly = 58.8514			
	k-mer completeness			98.187		75.5469	
				Full assembly = 99.6329			
	BUSCO completeness (mammalia_odb10) *n* = 9226		*C* ^c^	*S* ^c^	*D* ^c^	*F* ^c^	*M* ^c^
		*P* ^d^	96.30%	95.60%	0.70%	1.10%	2.60%
		*A* ^d^	62.60%	58.00%	4.60%	7.80%	29.60%
	Organelles		1 Partial mitochondrial sequence			JANIGQ010000317.1	

^a^Assembly quality code *x*.*y*.*P*.*Q*.*C* derived notation, from Rhie et al. (2021). *x* = log10[contig NG50]; *y* = log10[scaffold NG50]; *P* = log10 [phased block NG50]; *Q* = Phred base accuracy QV (quality value); *C* = % genome represented by the first “*n*” scaffolds, following a known karyotype for *U. amerianus* of 2*n* = 74 (Nash and O’Brien 1987). Quality code metrics were calculated from the primary assembly (mUrsAme1.0.p).

^b^Read coverage and NGx statistics have been calculated based on the estimated genome size of 2.37 Gb.

^c^BUSCO Scores. Complete BUSCOs (*C*). Complete and single-copy BUSCOs (*S*). Complete and duplicated BUSCOs (*D*). Fragmented BUSCOs (*F*). Missing BUSCOs (*M*).

^d^(P)rimary and (A)lternate assembly values.

Our assembly shows improved contiguity compared to other available assemblies for black bears (Table 3). Our primary assembly is represented in fewer contigs and scaffolds and has higher N50 statistics. The BUSCO scores for both our primary assembly and a previously assembled genome (GCF_020975775.1) are >95%, indicating that both assemblies are complete and single copies in these conserved regions of the genome.

**Table 3.** Comparison of genome assembly statistics.

	mUrsAme1.0.p	mUrsAme1.0.a	ASM334442v1	gsc_jax_bbear_1
NCBI Accession	GCA_024610735.1	GCA_024610745.1	GCA_003344425.1	GCF_020975775.1
Number of contigs	339	77,310	101,411	2,213
Contig N50 (bp)	58,859,121	43,280	190,236	13,882,922
Contig NG50^a^	59,189,856	60,742	210,302	13,882,922
Longest Contigs	107,133,695	831,372	2,352,914	95,818,817
Number of scaffolds	316	77,310	374,624	2,213
Scaffold N50	67,550,933	43,280	11,835	13,882,922
Scaffold NG50^a^	68,367,985	60,742	12,107	13,882,922
Largest scaffold	122,379,270	831,372	141,485	95,818,817
Size of final assembly	2,524,264,886	2,885,111,500	2,584,460,632	2,351,964,450
Gaps per Gbp (# Gaps)	9 (22)	0 (0)	144,952 (353,480)	0 (0)
BUSCO Scores (mammalia, *n* = 9,226)				
*C* ^b^	96.30%	62.60%	85.20%	95.90%
*S* ^b^	95.60%	58.00%	84.40%	95.30%
*D* ^b^	0.70%	4.60%	0.80%	0.60%
*F* ^b^	1.10%	7.80%	6.00%	1.20%
*M* ^b^	2.60%	29.60%	8.80%	2.90%

^a^NGx statistics calculated with an estimated genome size of 2.37 Gbp.

^b^BUSCO Scores. Complete BUSCOs (*C*). Complete and single-copy BUSCOs (*S*). Complete and duplicate BUSCOs (*D*). Fragmented BUSCOs (*F*). Missing BUSCOs (*M*).

We examined chromosome assignments and determined that our assembly is near-chromosome level. We identified scaffold JANIGQ010000001.1 in our assembly as the X chromosome based on synteny with the domestic dog genome. No scaffolds in our assembly had alignments to the domestic dog Y chromosome that matched our criteria of longer than 10 kb with greater than 80% identity. A handful of scaffolds had shorter alignments, indicating that the Y chromosome in our assembly is fragmented. We did not attempt to assign scaffolds in our assembly to autosomes based on the black bear karyotype (Nash and O’Brien 1987). However, we note that with a karyotype indicating 2*n* = 74 chromosomes, 92% of our assembly is contained in the 37 largest scaffolds (with the largest scaffold identified as the X chromosome), suggesting our assembly is near-chromosome level.

The final mitochondrial sequence has a size of 16,789 bp, with the base composition of *A* = 31.21%, *C* = 25.09%, *G* = 15.44%, *T* = 28.26%, and consisting of 2 rRNAs, 23 unique transfer RNAs, and 13 protein-coding genes. We evaluate the mitochondrial genome to be partial because while it is close to the expected size, the expected circularity is not supported. Additionally, while we annotated the expected number of rRNAs and protein-coding genes, the number of transfer RNAs differs from expected. The mitochondrial genome is scaffolded JANIGQ010000317.1 in our assembly.

## Discussion

We generated a high-quality, near chromosome-level genome assembly for an American black bear from California. This new genome will serve as the foundation for landscape and population genomic analyses that will aid conservation decision-makers. Large mammals can serve as umbrella species, whose conservation can extend protections to other species in the same habitat, and healthy bear populations are often an indication of ecosystem health (Pelton et al. 1999). The genome assembly is a foundational component of studies on the effects of habitat loss and fragmentation on wildlife populations, particularly the impacts of local adaptation and inbreeding depression.

This new black bear genome assembly expands opportunities for pangenomic analyses within the species. Both previously assembled genomes are from the eastern United States, whereas our new genome is from the western United States, enabling comparisons to identify potentially adaptive genomic differences to different habitats and anthropogenic pressures. For example, it is known that hibernation length and coat color vary across the range of black bears (Gámez-Brunswick and Rojas-Soto 2020; Puckett et al. 2023).

This new black bear genome assembly also expands opportunities for comparative genomic analyses among bear species. There are 8 extant species of bears, and all of them have high-quality reference genomes available or in progress (Willey and Korstanje 2022; Beth Shapiro, personal communication). These bear species live in diverse habitats from the Arctic to the Tropics and survive on a variety of diets, including both generalist and specialist diets (Pelton et al. 1999). The availability of genome assemblies for species with divergent ecological pressures and phenotypes enables the identification of both coding and regulatory variation that may underlie ecologically important variation. The inclusion of additional individuals and/or species into taxonomically broad multi-species alignments, such as the Zoonomia alignment of placental mammals (Christmas et al. 2023), may be useful in identifying adaptations unique to bears, in addition to functional variation that may be important for black bear conservation.

## Supplementary material

Supplementary material is available at *Journal of Heredity* Journal online.

esae037_suppl_Supplementary_Figure

## Data Availability

Data generated for this study are available under NCBI BioProject PRJNA777227. Raw sequencing data for sample L20-20 (NCBI BioSample SAMN29046565) are deposited in the NCBI Short Read Archive (SRA) under experiment accessions SRX17388741 (PacBio HiFi sequencing data) and SRX23638327-SRX23638328 (Omni-C Illumina sequencing data). GenBank accessions for the assemblies are GCA_024610735.1 (primary) and GCA_024610745.1 (alternate), with nucleotide sequences under accessions JANIGQ000000000 and JANIGR000000000, respectively. The partial mitochondrial nucleotide sequence can be found with GenBank accession JANIGQ010000317.1. Assembly workflow is available at www.github.com/ccgproject/ccgp_assembly.

## References

[CIT0001] Abdennur N , Mirny LA. Cooler: scalable storage for Hi-C data and other genomically labeled arrays. Bioinformatics. 2020:36:311–316. doi:10.1093/bioinformatics/btz540 31290943 PMC8205516

[CIT0002] Allio R , Schomaker-Bastos A, Romiguier J, Prosdocimi F, Nabholz B, Delsuc F. MitoFinder: efficient automated large-scale extraction of mitogenomic data in target enrichment phylogenomics. Mol Ecol Resour. 2020:20:892–905. doi:10.1111/1755-0998.13160 32243090 PMC7497042

[CIT0003] California Department of Fish and Game. Black Bear Management Plan. 1998. [Accessed 14 February 2024]. https://nrm.dfg.ca.gov/FileHandler.ashx?DocumentID=82769&inline

[CIT0004] Camacho C , Coulouris G, Avagyan V, Ma N, Papadopoulos J, Bealer K, Madden TL. BLAST+: architecture and applications. BMC Bioinf. 2009:10:421. doi:10.1186/1471-2105-10-421 PMC2803857 20003500

[CIT0005] Challis R , Richards E, Rajan J, Cochrane G, Blaxter M. BlobToolKit—interactive quality assessment of genome assemblies. G3 Genes Genomes Genetics. 2020:10:1361–1374. doi:10.1534/g3.119.400908 32071071 PMC7144090

[CIT0006] Cheng H , Jarvis ED, Fedrigo O, Koepfli K-P, Urban L, Gemmell NJ, Li H. Haplotype-resolved assembly of diploid genomes without parental data. Nature Biotechnology. 2022:40:1332–1335. doi:10.1038/s41587-022-01261-x PMC9464699 35332338

[CIT0007] Christmas MJ , Kaplow IM, Genereux DP, Dong MX, Hughes GM, Li X, Sullivan PF, Hindle AG, Lindblad-Toh K, Karlsson EK; Zoonomia Consortium. Evolutionary constraint and innovation across hundreds of placental mammals. Science. 2023:380:eabn3943. doi:10.1126/science.abn3943 37104599 PMC10250106

[CIT0008] Danecek P , Bonfield JK, Liddle J, Marshall J, Ohan V, Pollard MO, Whitwham A, Keane T, McCarthy SA, Davies RM, et al. Twelve years of SAMtools and BCFtools. GigaScience. 2021:10:giab008. doi:10.1093/gigascience/giab008 33590861 PMC7931819

[CIT0009] Delisle I , Strobeck C. Conserved primers for rapid sequencing of the complete mitochondrial genome from carnivores, applied to three species of Bears. Mol Biol Evol. 2002:19:357–361. doi:10.1093/oxfordjournals.molbev.a004090 11861896

[CIT0010] Gámez-Brunswick C , Rojas-Soto O. The effect of seasonal variation on the activity patterns of the American black bear: an ecological niche modeling approach. Mammalia. 2020:84:315–322. doi:10.1515/mammalia-2019-0017

[CIT0011] Ghurye J , Pop M, Koren S, Bickhart D, Chin C-S. Scaffolding of long read assemblies using long range contact information. BMC Genomics. 2017:18:527. doi:10.1186/s12864-017-3879-z 28701198 PMC5508778

[CIT0012] Ghurye J , Rhie A, Walenz BP, Schmitt A, Selvaraj S, Pop M, Phillippy AM, Koren S. Integrating Hi-C links with assembly graphs for chromosome-scale assembly. PLoS Comput Biol. 2019:15:e1007273. doi:10.1371/journal.pcbi.1007273 31433799 PMC6719893

[CIT0013] Gurevich A , Saveliev V, Vyahhi N, Tesler G. QUAST: quality assessment tool for genome assemblies. Bioinformatics. 2013:29:1072–1075. doi:10.1093/bioinformatics/btt086 23422339 PMC3624806

[CIT0014] Kerpedjiev P , Abdennur N, Lekschas F, McCallum C, Dinkla K, Strobelt H, Luber JM, Ouellette SB, Azhir A, Kumar N, et al. HiGlass: web-based visual exploration and analysis of genome interaction maps. Genome Biol. 2018:19:125. doi:10.1186/s13059-018-1486-1 30143029 PMC6109259

[CIT0015] Korlach J , Gedman G, Kingan SB, Chin C-S, Howard JT, Audet J-N, Cantin L, Jarvis ED. De novo PacBio long-read and phased avian genome assemblies correct and add to reference genes generated with intermediate and short reads. GigaScience. 2017:6:gix085. doi:10.1093/gigascience/gix085 PMC5632298 29020750

[CIT0016] Kurtz S , Phillippy A, Delcher AL, Smoot M, Shumway M, Antonescu C, Salzberg SL. Versatile and open software for comparing large genomes. Genome Biol. 2004:5:R12. doi:10.1186/gb-2004-5-2-r12 14759262 PMC395750

[CIT0017] Lee S , Bakker CR, Vitzthum C, Alver BH, Park PJ. Pairs and Pairix: a file format and a tool for efficient storage and retrieval for Hi-C read pairs. Bioinformatics. 2022:38:1729–1731. doi:10.1093/bioinformatics/btab870 34978573 PMC10060703

[CIT0018] Li H. 2013. Aligning sequence reads, clone sequences and assembly contigs with BWA-MEM. arXiv, arXiv:1303.3997. http://arxiv.org/abs/1303.3997, preprint: not peer reviewed.

[CIT0019] Manni M , Berkeley MR, Seppey M, Simão FA, Zdobnov EM. BUSCO update: novel and streamlined workflows along with broader and deeper phylogenetic coverage for scoring of eukaryotic, prokaryotic, and viral genomes. Mol Biol Evol. 2021:38:4647–4654. doi:10.1093/molbev/msab199 34320186 PMC8476166

[CIT0020] Nash WG , O’Brien SJ. A comparative chromosome banding analysis of the Ursidae and their relationship to other carnivores. Cytogenet Cell Genet. 1987:45:206–212. doi:10.1159/000132455 3691188

[CIT0021] Open2C, Abdennur N, Fudenberg G, Flyamer IM, Galitsyna AA, Goloborodko A, Imakaev M, Venev SV. Pairtools: from sequencing data to chromosome contacts. 2023. bioRxiv 2023.02.13.528389. 10.1101/2023.02.13.528389, preprint: not peer reviewed. PMC11164360 38809952

[CIT0022] Pelton MR , Coley AB, Eason TH, Doan Martinez DL, Pederson JA, van Manen FT, Weaver KM. American Black Bear Conservation Action Plan. In: Servheen C, Herrero S, Peyton B, editors. Bears: Status Survey and Conservation Action Plan. Cambridge, UK: IUCN; 1999. p. 144–156.

[CIT0023] Pflug JM , Holmes VR, Burrus C, Johnston JS, Maddison DR. Measuring genome sizes using Read-Depth, k-mers, and flow cytometry: methodological comparisons in beetles (Coleoptera). G3 (Bethesda, Md.). 2020:10:3047–3060. doi:10.1534/g3.120.401028 32601059 PMC7466995

[CIT0024] Puckett EE , Davis IS, Harper DC, Wakamatsu K, Battu G, Belant JL, Beyer DE, Carpenter C, Crupi AP, Davidson M, et al. Genetic architecture and evolution of color variation in American black bears. Curr Biol. 2023:33:86–97.e10. doi: 10.1016/j.cub.2022.11.042 36528024 PMC10039708

[CIT0025] Ramírez F , Bhardwaj V, Arrigoni L, Lam KC, Grüning BA, Villaveces J, Habermann B, Akhtar A, Manke T. High-resolution TADs reveal DNA sequences underlying genome organization in flies. Nat Commun. 2018:9:189. doi:10.1038/s41467-017-02525-w 29335486 PMC5768762

[CIT0026] Ranallo-Benavidez TR , Jaron KS, Schatz MC. GenomeScope 2.0 and Smudgeplot for reference-free profiling of polyploid genomes. Nat Commun. 2020:11:1432. doi:10.1038/s41467-020-14998-3 32188846 PMC7080791

[CIT0027] Rhie A , McCarthy SA, Fedrigo O, Damas J, Formenti G, Koren S, Uliano-Silva M, Chow W, Fungtammasan A, Kim J, et al. Towards complete and error-free genome assemblies of all vertebrate species. Nature. 2021:592:737–746. doi:10.1038/s41586-021-03451-0 33911273 PMC8081667

[CIT0028] Rhie A , Walenz BP, Koren S, Phillippy AM. Merqury: reference-free quality, completeness, and phasing assessment for genome assemblies. Genome Biol. 2020:21:245. doi:10.1186/s13059-020-02134-9 32928274 PMC7488777

[CIT0029] Shaffer HB , Toffelmier E, Corbett-Detig RB, Escalona M, Erickson B, Fiedler P, Gold M, Harrigan RJ, Hodges S, Luckau TK, et al. Landscape genomics to enable conservation actions: The California Conservation Genomics Project. J Hered. 2022:113:577–588. doi:10.1093/jhered/esac020 35395669

[CIT0030] Sim SB , Corpuz RL, Simmonds TJ, Geib SM. HiFiAdapterFilt, a memory efficient read processing pipeline, prevents occurrence of adapter sequence in PacBio HiFi reads and their negative impacts on genome assembly. BMC Genomics. 2022:23:157. doi:10.1186/s12864-022-08375-1 35193521 PMC8864876

[CIT0031] Srivastava A , Kumar Sarsani V, Fiddes I, Sheehan SM, Seger RL, Barter ME, Neptune-Bear S, Lindqvist C, Korstanje R. Genome assembly and gene expression in the American black bear provides new insights into the renal response to hibernation. DNA Res. 2019:26:37–44. doi:10.1093/dnares/dsy036 30395234 PMC6379037

[CIT0032] Uliano-Silva M , Ferreira JGRN, Krasheninnikova K, Formenti G, Abueg L, Torrance J, Myers EW, Durbin R, Blaxter M, McCarthy SA; Darwin Tree of Life Consortium. MitoHiFi: a python pipeline for mitochondrial genome assembly from PacBio high fidelity reads. BMC Bioinf. 2023:24:288. doi:10.1186/s12859-023-05385-y PMC10354987 37464285

[CIT0033] Wilder AP , Supple MA, Subramanian A, Mudide A, Swofford R, Serres-Armero A, Steiner C, Koepfli K-P, Genereux DP, Karlsson EK, et al; Zoonomia Consortium‡. The contribution of historical processes to contemporary extinction risk in placental mammals. Science. 2023:380:eabn5856. doi:10.1126/science.abn5856 37104572 PMC10184782

[CIT0034] Willey C , Korstanje R. Sequencing and assembling bear genomes: the bare necessities. Front Zool. 2022:19:30. doi:10.1186/s12983-022-00475-8 36451195 PMC9710173

