# Supplementary material for: A genome assembly of the American black bear, Ursus americanus, from California
Source: J Hered. 2024 Jul 15;115(5):498–506. doi: 10.1093/jhered/esae037 (PMC11334205; doi:10.1093/jhered/esae037)
Supplement: esae037_suppl_Supplementary_Figure [file esae037_suppl_supplementary_figure.docx]

**
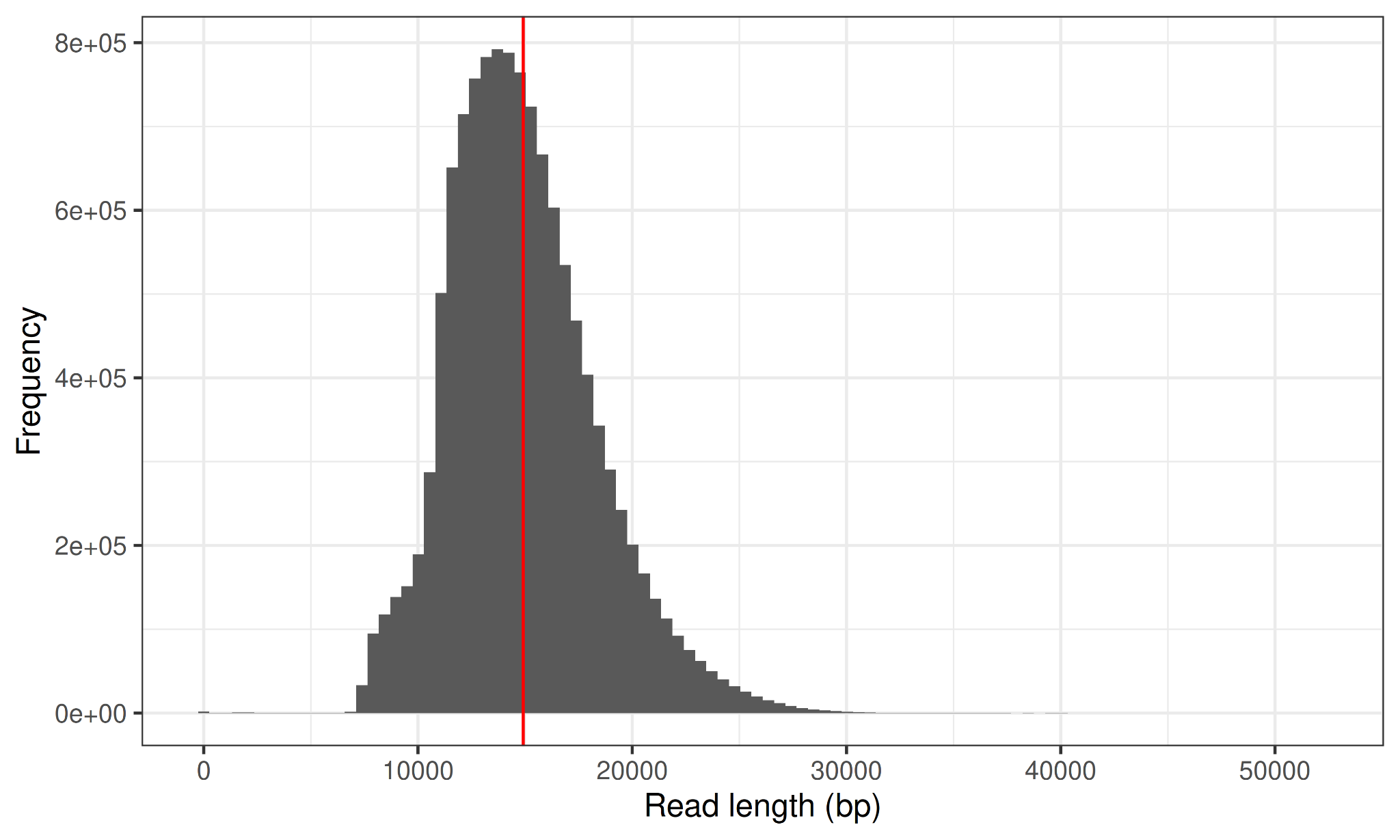
**

**Supplementary Figure S1.** Read length distribution of PacBio HiFi reads. The red line indicates the average read length.
